# Diagnosing Schwannoma in the Wrist: a Challenging Case of Intraneural Ganglion Cyst Mimicry and a Review of Literature

**DOI:** 10.1080/23320885.2023.2249099

**Published:** 2023-08-26

**Authors:** Hatan Mortada, Abdulaziz M. Alghamdi, Abdulmuhsen N. Alshammari, Saad Alrobaiea, Musab Alrehaili, Abdullah Kattan

**Affiliations:** a Division of Plastic Surgery, Department of Surgery, King Saud University Medical City, King Saud University, Riyadh, Saudi Arabia; b Department of Plastic Surgery & Burn Unit, King Saud Medical City, Riyadh, Saudi Arabia; c College of Medicine, King Saud bin Abdulaziz University for Health Sciences, Jeddah, Saudi Arabia; d Department of Orthopedics, King Salman Bin Abdulaziz Medical City, Almadinah Almunawwarah, Saudi Arabia; e Department of Plastic Surgery and Burn Unit, Security Forces Hospital, Riyadh, Saudi Arabia; f Division of Plastic Surgery, Department of Surgery, College of Medicine, King Saud University, Riyadh, Saudi Arabia

**Keywords:** Schwannoma, intraneural ganglion cyst, pitfalls, hand surgery, case report

## Abstract

Schwannoma can rarely mimic an intraneural ganglion cyst clinically and radiographically. This is a rare case report of a wrist schwannoma mimicking an intraneural ganglion cyst. The surgery was successful, and the histopathological report confirmed the diagnosis of benign schwannoma. After two years of follow-up, the patient is still symptom-free.

## Introduction

Schwannoma is the most benign peripheral nerve tumor originating from Schwann cells of the nerve sheath. Schwannomas are usually isolated, solitary, slow-growing, and encapsulated neoplasms composed totally of Schwann cells of myelin sheaths. Although they can occur at any age and in any part of the body, they most commonly tend to occur between the third and sixth decades of age and appear in the head and neck areas of the body. Also, there is no sex predisposition that appears to exist for the occurrence of these tumors [1,2]. In clinical practice, Ultrasonography and/or Magnetic Reasoning Imaging (MRI) can help in the diagnosis. On ultrasound, these lesions usually present as a homogenous, hypoechogenic, round mass. On MRI, they usually present as a round, hyperintense to fat, homogeneous mass, with a poorly enhanced central part and enhanced peripheral part, which is known as the target sign. However, small schwannomas can present without a distinguishing target sign, which in turn can make them mimic cysts in appearance [1,3]. With that being said, small schwannomas can be misdiagnosed as intraneural ganglion cysts if they are presented in the hand or wrist, which is the most common site for ganglion cysts to occur [3]. We present a case report of a patient with a wrist schwannoma mimicking an intraneural ganglion cyst, and we aimed to highlight the potential diagnostic challenges in distinguishing schwannoma from intraneural ganglion cyst in unusual locations, such as the wrist. We also provided a comprehensive review of the clinical, radiological, and pathological features of similar cases reported in the literature.

## Case study

A 43-year-old male patient, with no significant medical or surgical history, presented to the clinic with two painful lesions located on the dorsum of his right wrist. The patient first noticed these lesions three years ago, and they have been a source of persistent pain and discomfort. The patient reported occasional hand pain, but denied any numbness, tingling, functional impairment, or weakness. There was no history of trauma, infection, or constitutional symptoms. A thorough review of the patient’s medical history did not reveal any notable findings, and there was no family history of neurofibromatosis or similar conditions.

Upon examination, two painful subcutaneous palpable lesions were identified on the dorsum of the right wrist. These lesions exhibited an oval shape and demonstrated non-mobility, non-pulsation, and soft consistency. Each lesion measured less than 10 mm in size and appeared nearly identical in dimensions.

**Figure 1.**
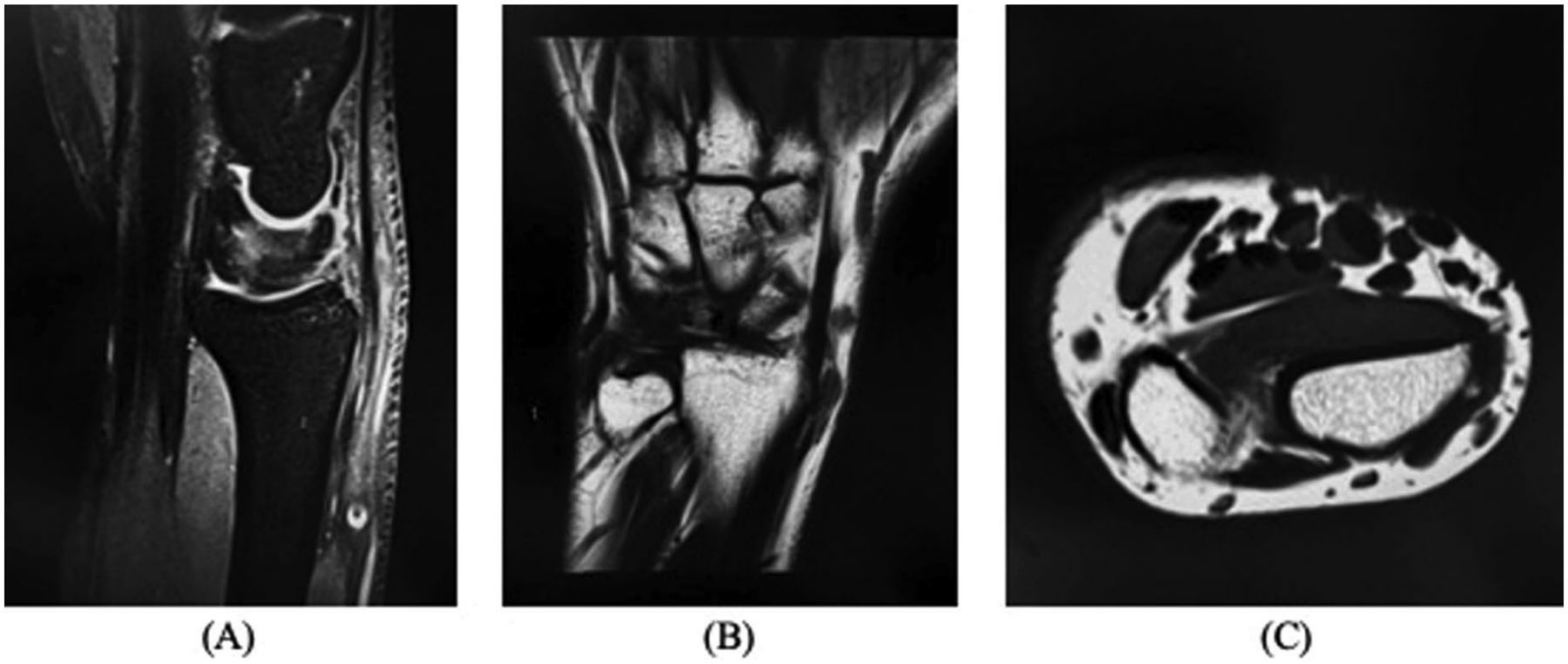
MRI image of a wrist Schwannoma mimicking an intraneural ganglion cyst. A well-defined ovoid structure lateral to the extensor carpi radialis tendon, measuring 6 x 5 mm and communicating with the underlying joint space. (A) Sagittal, (B) Coronal, and (C) Transverse view.

Tinel’s and Phalen’s signs were negative. All motor and sensory examinations of the hand were normal. To diagnose the lesions, he underwent both hand ultrasound and MRI, and the radiological characteristics were consistent with cystic lesions (Figure 1).

**Figure 2.**
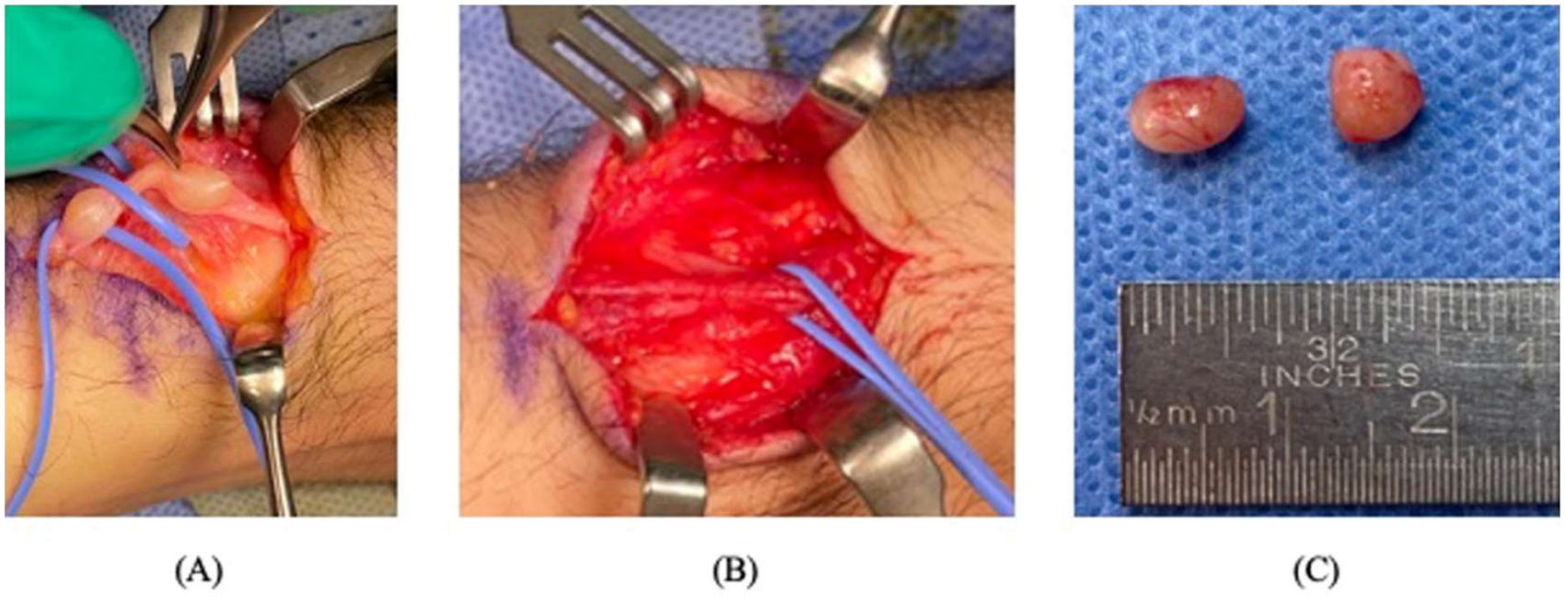
An intraoperative image of a schwannoma in the wrist shows a well-circumscribed tumor located on the superficial radial nerve. The image was obtained during the surgical procedure for excision of the tumor. Panel (A) shows two separate, well-defined oval masses adhered to the superficial radial nerve. Panel (B) shows the nerve after excision of both tumors, as the nerve showed continuity without disruption. (C) shows a gross picture of both lesions after removal.

**Figure 3.**
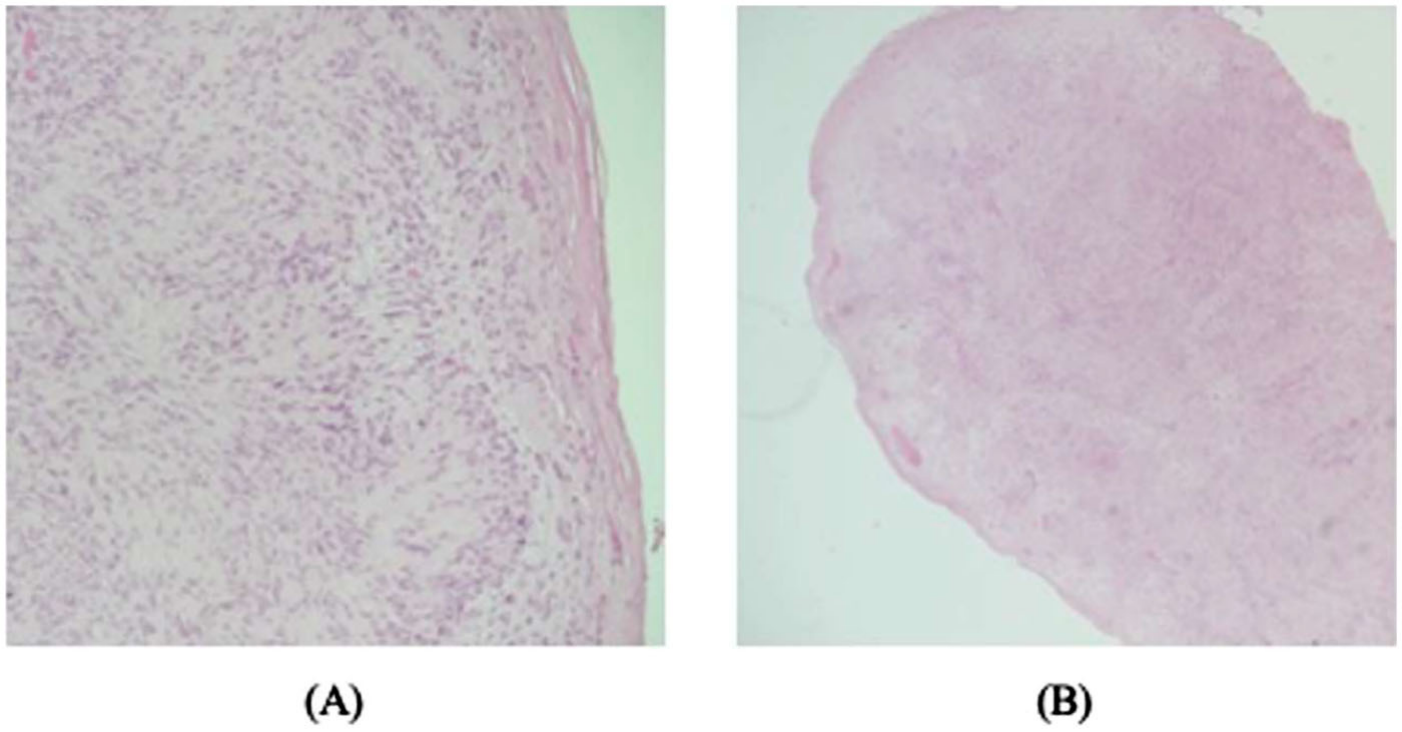
Typical schwannoma with palisaded wavy nuclei on a myxoid background, without mitotic activity (hematoxylin-eosin, 1003).

During the ultrasound examination, the two lesions exhibited distinct morphological and structural characteristics. The ultrasound findings revealed that both lesions appeared as cystic structures. They exhibited well-defined boundaries and were ovoid in shape. The lesions were located laterally to the extensor carpi radialis tendon. The MRI without contrast report showed two separate, well-defined ovoid structures located laterally to the extensor carpi radialis tendon. The largest of these structures measured 6 × 5 mm and communicated with the underlying joint space, consistent with a ganglion cyst. A preoperative diagnosis of two intraneural ganglion cysts was made, and after discussing treatment options, he was scheduled for elective surgery to remove the lesions. In the operating room, when the skin was cut open, and the lesions were first exposed, they exhibited a gross appearance that was more consistent with Schwannoma tumors, with no cystic material emerging on aspiration (Figure 2). Both lesions were excised and sent to the histopathology department for correlation of the preoperative diagnosis and the histopathological features, and the surgery was successful without complications. Two Schwannoma tumors were diagnosed based on their histopathological characteristics (Figure 3). The patient was symptom-free in the clinic for his 2-year follow-up appointment without any recurrence of symptoms or lesions. Written informed consent was obtained from the patient for the publication of this case report, including the use of images and other relevant medical information. The patient was informed of the purpose of the publication, the potential risks of confidentiality breaches, and the measures taken to ensure anonymity. The patient was given the opportunity to ask questions and had their concerns addressed before providing consent.

## Discussion

This is a rare case report of a benign wrist schwannoma tumor that was initially misdiagnosed by its clinical presentation and MRI features as an intraneural ganglion cyst.

The literature review revealed ten similar case reports of upper-extremity schwannomas that were initially misdiagnosed as ganglion cysts (Table 1) [4–12].

This study employed a narrative research strategy to identify relevant articles for inclusion in the literature review. The search was conducted using two databases, including PubMed and Google Scholar. The search terms used were ‘schwannoma’, ‘intraneural ganglion cyst’, ‘wrist’, and ‘mimic’. The inclusion criteria for article selection were as follows: (1) Articles published in English language, (2) Case reports or case series describing a wrist schwannoma mimicking an intraneural ganglion cyst, (3) Detailed clinical and radiographic presentation, (4) Surgical intervention and histopathological confirmation of the diagnosis, (5) Adequate follow-up to assess patient outcomes. The exclusion criteria were: (1) Articles not directly related to the specific case of wrist schwannoma mimicking an intraneural ganglion cyst, (2) Articles lacking essential clinical or radiographic information, (3) Studies with incomplete or insufficient follow-up data.

The enrolled patients’ ages ranged from 22 to 70 years old, and out of the 10 cases, 7 were females. The presenting symptoms were pain, paresthesia, and/or weakness, and mostly, the symptoms were chronic in nature. No specific patterns were noticed in the tumors’ locations, as they can present anywhere along the nerves’ courses. Preoperative ultrasound and/or MRI were done in 9 out of 10 cases, and they all were consistent with ganglion cysts [4–12]. The postoperative histopathological diagnoses in all the cases were schwannomas; in most cases, there were no complications after the surgery. Two cases reported postoperative complications of paresthesia and/or weakness in the affected nerve distributions [7,11]. However, in the follow-up, they had normal functional status with no recurrence, just like most of the other reported cases, showing a favorable prognosis [4,6,7,9–12].

Schwannomas, as mentioned earlier, are the most common benign peripheral nerve tumors. However, their occurrence in the upper extremities is rare compared to other neoplasms [1]. They grow inside nerves, causing a thickening of the nerve and a visible lump underneath the skin. In the early stage, the subcutaneous lesions are usually painless. However, large and long-growing lesions may cause pain, paresthesia, and weakness [1,2]. Unlike benign schwannoma, malignant ones grow faster and cause more pronounced symptoms. Fortunately, they are extremely rare [1]. Schwannomas are characterized by their slow growth, and they can undergo degeneration such as cyst formation, fibrosis and calcification, in a case report by Valeriu Ardeleanu et al. a cystic lesion in the upper extremity was radiologically and intraoperatively identified, and its histopathological report was diagnostic of a completely cystic schwannoma [1,4]. Both ultrasound and MRI should be used to diagnose schwannomas [2]. According to Gregory Scott Stacy et al. schwannomas can appear hypoechoic with variable posterior acoustic enhancement on ultrasound, and hyperintense to fat on T2-weighted MRI, thereby mimicking cysts radiologically, especially when they are small and homogeneous. The two radiologically characteristic signs of schwannoma ‘tail sign’ and ‘target sign’, which refer to the entering and exiting nerve and the poorly enhanced central part with enhanced peripheral part, respectively, should be seen to help in the differentiation of schwannomas from the other types of cysts, however, they may not be seen with smaller nerves in hand [3]. One of the common intraneural cysts that can be confused with schwannomas is the intraneural ganglion cyst [4–13]. *Intraneural ganglion cysts* are defined as mucinous non-neoplastic cysts within the epineurium of a nerve and commences from an adjoining joint. Their clinical presentation and radiological features can mimic schwannomas, specifically when it is affecting the wrists or the knees [3,13]. However, the surgical treatment of cystic schwannomas is enucleation instead of intraneural ganglion cysts, where the nerve is decompressed, and the articular branch is excised [13]. Both careful planning and accurate diagnosis before operation are helpful in dealing with schwannoma and intraneural ganglion cyst cases to ensure optimum efficiency of nerve recovery and to reduce unnecessary damage to nerve fascicles. The prognosis of such tumors and cysts is favorable, and they are usually curative after surgical removal [2,13].

This study highlights and re-emphasizes the importance of keeping benign schwannoma as a differential diagnosis of upper extremity radiologically cystic-appearing lesions. This study, as mentioned, is a case report and literature review, which may impact the reliability and the strength of evidence of the data. A larger retrospective study focusing on the differentiation of both lesions are recommended.

## Conclusion

Schwannoma and intraneural ganglion cyst can present in a clinically and radiologically similar manner. As a result, maintaining a differential diagnosis of both lesions and carefully planning surgical management is essential. The prognosis of both lesions is generally favorable. Surgeons and radiologists should collaborate in the preoperative diagnosis to minimize misdiagnosis rates and associated complications. Although schwannoma mimicking intraneural ganglion cysts is a rare presentation that poses a significant diagnostic challenge, clinicians should maintain a high index of suspicion and thoroughly evaluate all diagnostic data to avoid misdiagnosis and ensure proper treatment. Increased awareness of this entity may result in a more timely diagnosis, better patient outcomes, and an improved quality of life.

## Figures and Tables

**Table 1.** Synopsis of the similar case reports in the literature.

First author, year	Sex	Age	Presenting symptoms					Duration of the symptoms	Tumor location	Size	Nerve involved
			Tinel’s sign	Phalen’s sign	Pain	Weakness	Paresthesia				
Valeriu Ardeleanu et al. 2020 [4]	Female	33	NM	NM	Yes	No	No	NM	The left distal forearm	2.5 × 3 cm	NM
Mooi SS et al. 2016 [5]	Female	32	NM	NM	No	No	No	6 months	The dorsal surface of the right thumb	2 × 2 cm	NM
Vuk Aleksić et al. 2021 [6]	Female	22	Positive	NM	No	No	No	several years	The dorsal region of the left hand	3 × 2 cm	Ulnar nerve
Arshad M. Bhatti et al. 2005 [7]	Female	59	Negative	NM	No	No	No	8 months	The volar wrist on the arm	3 × 8 cm	Median nerve
Ganesh Singh Dharmshaktu et al. 2019 [8]	Female	38	NM	NM	No	No	No	3 years	The medial aspect of the left index finger	1 × 0.5 cm	NM
Angela Lim et al. 2012 [9]	Male	70	NM	NM	Yes	NM	NM	3 years	Deep to the flexor tendons at the level of the metacarpals	NM	Ulnar nerve
Ahmadreza Afshar, 2014 [10]	Male	30	Positive	Negative	Yes	Yes	Yes	1 year	The left hand’s antecubital fossa	5 × 3 cm	Median nerve
N. Aslam et al. 2003 [11]	Male	36	Positive	Positive	Yes	Yes	Yes	2 years	The palm of the right hand	1 × 0.8 cm	Median nerve
H. J. Kang et al. 2016 [12]	Female	22	No	NM	NM	Yes	No	NM	The right wrist	2 × 2 cm	Ulnar nerve
Panwar, 2017 [13]	Female	48	Positive	NM	NM	No	No	NM	The right wrist	2 × 3 cm	Ulanr nerve

First author, year	Preoperative ultrasound	Preoperative MRI	Preoperative diagnosis	Histopathological diagnosis	Outcome	Follow up period	prognosis
Valeriu Ardeleanu et al. 2020 [4]	Yes	No	Ganglion cyst	Cystic schwannoma	Favorable	10 months	Normal functional status with no recurrence
Mooi SS et al. 2016 [5]	Yes	No	Ganglion cyst	Schwannoma	Favorable	NM	NM
Vuk Aleksić et al. 2021 [6]	Yes	No	Ganglion cyst or schwannoma	Schwannoma	Favorable	3 months	Normal functional status with no recurrence
Arshad M. Bhatti et al. 2005 [7]	No	Yes	Ganglion cyst	Schwannoma	Paresthesia in the median nerve distribution	18 months	Normal functional status with no recurrence
Ganesh Singh Dharmshaktu et al. 2019 [8]	Yes	No	Ganglion cyst	Plexiform Schwannoma	NM	NM	NM
Angela Lim et al. 2012 [9]	No	Yes	Ganglion cyst	Schwannoma	Favorable	10 weeks	Normal functional status with no recurrence
Ahmadreza Afshar, 2014 [10]	Yes	Yes	Ganglion cyst or schwannoma	Schwannoma	Favorable	6 months	Normal functional status with no recurrence
N. Aslam et al. 2003 [11]	No	Yes	Ganglion cyst	Schwannoma	Dysaethesia in the median nerve distribution with associated weakness of thumb opposition	6 months	Normal functional status with no recurrence
H. J. Kang et al. 2016 [12]	No	Yes	Ganglion cyst	Schwannoma	Favorable	> 18 months	Normal functional status with no recurrence
Panwar, 2017 [13]	No	No	Ganglion cyst	Schwannoma	Favorable	> 18 months	Normal functional status with no recurrence
